# Costs of continuing RTS,S/ASO1E malaria vaccination in the three malaria vaccine pilot implementation countries

**DOI:** 10.1371/journal.pone.0244995

**Published:** 2021-01-11

**Authors:** Ranju Baral, Ann Levin, Chris Odero, Clint Pecenka, Collins Tabu, Evans Mwendo, George Bonsu, John Bawa, John Frederick Dadzie, Joyce Charo, Kwadwo Odei Antwi-Agyei, Kwame Amponsa-Achianou, Rose Eddah Jalango, Rouden Mkisi, Scott Gordon, Temwa Mzengeza, Winthrop Morgan, Farzana Muhib

**Affiliations:** 1 Center for Vaccine Innovation and Access, PATH, Seattle, Washington DC, United States of America; 2 Levin and Morgan LLC, Levin, Maryland, United States of America; 3 Center for Vaccine Innovation and Access, PATH, Nairobi, Kenya; 4 Expanded Program on Immunization, Ministry of Health, Nairobi, Kenya; 5 Expanded Program on Immunization, Ministry of Health, Lilongwe, Malawi; 6 Expanded Program on Immunization, Ministry of Health, Accra, Ghana; 7 Center for Vaccine Innovation and Access, PATH, Accra, Ghana; 8 Center for Vaccine Innovation and Access, PATH, Lilongwe, Malawi; Instituto Rene Rachou, BRAZIL

## Abstract

**Background:**

The RTS,S/ASO1_E_ malaria vaccine is being piloted in three countries—Ghana, Kenya, and Malawi—as part of a coordinated evaluation led by the World Health Organization, with support from global partners. This study estimates the costs of continuing malaria vaccination upon completion of the pilot evaluation to inform decision-making and planning around potential further use of the vaccine in pilot areas.

**Methods:**

We used an activity-based costing approach to estimate the incremental costs of continuing to deliver four doses of RTS,S/ASO1_E_ through the existing Expanded Program on Immunization platform, from each government’s perspective. The RTS,S/ASO1_E_ pilot introduction plans were reviewed and adapted to identify activities for costing. Key informant interviews with representatives from Ministries of Health (MOH) were conducted to inform the activities, resource requirements, and assumptions that, in turn, inform the analysis. Both financial and economic costs per dose, cost of delivery per dose, and cost per fully vaccinated child (FVC) are estimated and reported in 2017 USD units.

**Results:**

At a vaccine price of $5 per dose and assuming the vaccine is donor-funded, our estimated incremental financial costs range from $1.70 (Kenya) to $2.44 (Malawi) per dose, $0.23 (Malawi) to $0.71 (Kenya) per dose delivered (excluding procurement add-on costs), and $11.50 (Ghana) to $13.69 (Malawi) per FVC. Estimates of economic costs per dose are between three and five times higher than financial costs. Variations in activities used for costing, procurement add-on costs, unit costs of per diems, and allowances contributed to differences in cost estimates across countries.

**Conclusion:**

Cost estimates in this analysis are meant to inform country decision-makers as they face the question of whether to continue malaria vaccination, should the intervention receive a positive recommendation for broader use. Additionally, important cost drivers for vaccine delivery are highlighted, some of which might be influenced by global and country-specific financing and existing procurement mechanisms. This analysis also adds to the evidence available on vaccine delivery costs for products delivered outside the standard immunization schedule.

## Introduction

Malaria remains a major public health problem, with 228 million cases and more than 400,000 deaths worldwide in 2018 [1]. Most of this burden (about 94%) is concentrated in sub-Saharan Africa, and children under five years old are most vulnerable, contributing to 67% of all malaria deaths in 2018 [1].

The RTS,S/ASO1_E_ vaccine is the first malaria vaccine shown to provide partial protection against malaria in young children [2,3]. The World Health Organization (WHO) recognized RTS,S/ASO1_E_ as a potential complementary tool in the fight against the global malaria burden and recommended pilot implementation of the vaccine in three to five settings of moderate to high risk in sub-Saharan Africa [4]. Following the WHO recommendation, and as part of the coordinated evaluation of malaria vaccine introduction, three countries, Ghana, Kenya, and Malawi, started providing the malaria vaccine in selected areas as a pilot implementation program, beginning in 2019 [4]. As a part of the evaluation of vaccine introduction, selected areas in each country introduced the vaccine through the routine immunization system (henceforth referred to as implementation areas), while other regions/districts served as comparison areas. The four-dose RTS,S/ASO1_E_ vaccine is administered to all eligible children in the pilot implementation areas. Children are eligible for the first three doses in the first year of life, followed by a fourth dose at around two years old [4,5].

Funding agencies have supported the pilot introduction of RTS,S/ASO1_E_ [6], and GlaxoSmithKline (GSK), the vaccine’s manufacturer, has committed to donating up to 10 million doses of vaccine for pilot implementation. The economic implications of sustaining malaria vaccine delivery once the pilot ends need further exploration. Once immunization for the pilot is complete, donated vaccines and support for operational costs may no longer be available. Therefore, the cost of the vaccine and other operational costs may need to be financed through other mechanisms. Governments and other vaccine financing institutions will need to determine how to financially support the continued administration of the vaccine in implementation areas and how to scale-up the RTS,S/ASO1_E_ intervention in comparison areas. Understanding the costs associated with both introducing RTS,S/ASO1_E_ in comparison areas and continuing vaccination in intervention areas following the pilot will be critical in assessing the economics of sustaining this intervention and can help inform decision-making and planning around further use of the vaccine.

We aim to estimate the financial and economic incremental costs of continuing to provide the malaria vaccine in the pilot implementation areas and to introduce malaria vaccination in the comparison areas, through the routine Expanded Program on Immunization (EPI), in Ghana, Kenya, and Malawi. The estimates generated will inform country decision-making regarding the further use of RTS,S/ASO1_E_ and the economic implications of continuing vaccination after the pilot ends. The study also provides insights on how similar the cost of introducing RTS,S/ASO1_E_ is to other vaccines in the routine immunization system.

## Materials and methods

The costing analysis follows widely used guidelines in estimating the costs of new vaccine introduction and delivery [7,8]. We used an activity-based costing approach to estimate the incremental costs associated with continuing to provide the malaria vaccine through the existing EPI platform, from each government’s perspective. The analysis does not include cost to the patients or the broader societal cost of the intervention. All activities associated with the introduction and delivery of the malaria vaccine were identified and costed individually, using a Malaria Vaccine Introduction Costing tool (MVICT), an excel-based tool developed specifically for this purpose. The tool is similar to other WHO costing tools; for example, the Seasonal Influenza Immunization Costing Tool (SIICT) and Cervical Cancer Prevention and Control Costing Tool (C4P), that are developed to help countries plan and project the cost of introducing new vaccines. The MVICT was reviewed by WHO’s Immunization and Vaccine Implementation Research Advisory Committee (IVIR-AC) in September 2017. The tool is available upon request from the corresponding author.

The study team reviewed the malaria vaccine pilot introduction plans in each country to identify potential activities needed to introduce and continue vaccination in the pilot areas, following the pilot’s completion. Activities planned and implemented for the pilot were reviewed and discussed extensively within the study team before adapting and or/finalizing activities for the post-pilot introduction. Data on inputs and assumptions were agreed upon by the study team, which included EPI representatives from each country. Key informant interviews within the respective Ministries of Health (MOHs) at national and sub-national levels were conducted for additional information, as needed, to inform the analysis. Data collection occurred in 2017 in Ghana and in 2018 in Malawi and Kenya, with follow-up data collection in 2019. The analysis estimates costs for a seven-year time frame (2020–2026). The seven-year time horizon was used to include all children vaccinated with first dose in year 5 to allow time for them to complete all four doses as the fourth dose of vaccine is not delivered to children until the age of 2 years.

### Scope of cost analysis

This cost analysis was conducted in the context of the malaria vaccine pilot. Malaria vaccine implementation within the pilot was done in selected areas (regions/districts) in each country. Within the selected areas, some sub-regions introduced the vaccine into their immunization schedules, while others remained as comparison areas. Assignment of implementation areas and comparison areas was randomized by using a computer program, in consultation with each country’s EPI and National Malaria Control Program (NMCP).

We costed two distinct scenarios of vaccine implementation: continuing to vaccinate children within the implementation areas after the pilot vaccination ends (Scenario 1), and introduction of the vaccine in comparison areas while also continuing to vaccinate children within implementation areas (Scenario 2). For each scenario, we assess costs under alternative financial scenarios where each government pays 0%, 50%, 100% of direct vaccine-related costs—costs that are supported entirely by donor agencies during the pilot.

### Cost components

All activities identified for costing were grouped into key components of the vaccination program, including vaccine procurement, microplanning, training, communications, social mobilization, cold chain expansion, service delivery, supervision, and monitoring of vaccine delivery. Within each category, the levels and type of sub-activities vary across the countries. A detailed list of activities used for costing is included in the Supplementary material (S1 Table).

#### Procurement

Procurement included costs of purchasing vaccine and injection supplies. The number of doses or units of vaccine and injection supplies accounted for the probable wastage and buffer stock requirements. Costs associated with shipping, freight, handling, and clearance were added as a percentage of the base price of vaccine/injection supplies as procurement add-on costs. The cost of the vaccine was assumed to be $5 per dose. Procurement add-on costs are country-specific values and informed by current levels incurred for other vaccines in each country. In the baseline financial scenario, vaccines were assumed to be donated at no cost to the government, but with each government responsible for procurement add-on costs. Additional financial scenarios where governments pay 50% and 100% of vaccine procurement costs were also analyzed separately.

#### Distribution and storage (cold chain)

Distribution of vaccine and supplies included transportation from the national level store to the health facilities. The key informants indicated the frequency and mode of distribution at each level. The total amount of resources required for each distribution trip was estimated using the cost ingredients, which include fuel and per diems of drivers and other staff involved in the process. Spare capacity for distribution of vaccines was assumed with no additional capital investment. To account for the incremental resource requirements, distribution was valued as a shared input, apportioning a fraction of the distribution costs to the malaria vaccine, based on guidance from the EPI. Cold chain for vaccine storage and distribution were assumed at spare capacity in Kenya and Malawi. In Ghana, the purchase of additional cold chain equipment was identified and costed. Operational costs for cold chain maintenance were excluded from the analysis.

#### Microplanning

Microplanning activities entail activities for planning the introduction and delivery of the malaria vaccine at various levels. Costs of microplanning activities included per diems for participants and facilitators, conference packages, fuel and per diem allowances for travel, and other supplies as needed.

#### Training

Trainings were identified for staff at all levels and included a range of activities by country, such as refresher trainings for health workers already in the vaccine implementation areas (in some countries), and planning meetings and material development workshops. For each training session, the estimated cost was comprised of venue rentals, conference packages, per diems for participants and facilitators, and other supplies. The costing components were adapted from each country’s pilot plans [9–11].

#### Sensitization

Sensitization activities included briefing and orientation meetings for health workers, media personnel, professional groups, political and community leaders, and other stakeholders. Costs associated with sensitization varied by type of sub-activity, but broadly included the cost of supplies, per diem, conference packages, and reimbursements for fuel.

#### Social mobilization

Social mobilization included community-level activities geared toward educating communities on malaria vaccination through forums such as community meetings, radio talks, and volunteer mobilization. Costs included supplies, per diem, and coordination by health workers in facilitating such activities.

#### Communications

Communications included the costs of printing communications materials such as posters, job aides, and leaflets, as well as mass media broadcast materials. Costs associated with communications workshops were also categorized under communications and included allowances, venue rental, fuel, and other supplies.

#### Supervision, monitoring, and evaluation

The value of additional resources such as the modification and printing of program monitoring tools were included under monitoring and evaluation. Additionally, the supervision and monitoring for readiness assessments, and pre- and post- introduction evaluations were also included under this category. The pre- and post- introduction supervision and monitoring included the costs of facilitation from national and regional levels, per diems, fuel, drivers, and allowances for drivers. Routine supervision was considered a shared cost and attributed to RTS,S/ASO1_E_ based on direct allocation.

#### Service delivery

Service delivery components include costs associated with human resources for vaccine administration at health facilities. Such costs include administration of malaria vaccines during routine fixed clinic sessions, routine outreach sessions, mop-up campaigns, and other vaccination sessions. The EPI, in consultation with select health workers, estimated the time needed for each vaccination during these sessions, including preparation for vaccine administration, actual administration, and recording/reporting. Costs of vaccinations at outreach and campaign settings also included travel allowances, lunch allowances, and travel reimbursement for staff and volunteers involved in the delivery. Spare capacity was assumed for staff as no additional hiring was anticipated.

In adapting the malaria vaccine implementation plans, activities unique to pilot implementation and those related to the program evaluation component of the Malaria Vaccine Implementation Programme (MVIP) were excluded from this analysis to accurately reflect costs outside a pilot setting. Specifically, pharmacovigilance system strengthening in countries that did not have adequate systems established was a prerequisite of the pilot program and absorbed under the pilot costs. For the continuous vaccination post pilot, we excluded pharmacovigilance system strengthening costs as there would be no incremental costs associated with this activity post pilot. Costs of vaccine distribution was partly covered by the donor agencies during the pilot implementation. In this analysis, all costs of vaccine distribution were included and assume to be paid by the government to reflect routine vaccine implementation. Similarly, activities such as planning activities specifically relevant for the pilot program were excluded. Social mobilization and sensitization activities included in the pilot program were assumed to sustain through the continuous vaccination program.

Table 1 shows key data inputs and assumptions used for the analysis, particularly the demographic data, vaccine characteristics, vaccine utilization, service delivery, values, and sources. The demographic data were drawn from the pilot catchment areas in the countries and projected for the study duration, using country-specific population growth rates. All costs were categorized into recurrent and introduction costs as well as financial and economic costs (see Supplementary material, S2 Table).

**Table 1:** Key inputs and assumptions.

Categories	Malawi	Ghana	Kenya	Data source
**Demographics**				
Target population (Number of infants in designated intervention and comparison areas in year 2020)	286,585	338,539	310,636	MVIP country introduction plans [9–11]
Population growth rate	Average 3.1% [2.4% to 3.6%]	Average 2.5% [2.0% to 3.1%]	Constant at 2.60%	EPI
**Immunization program**				
Number of health facilities providing vaccinations	214	947	1,304	MVIP country introduction plans [9–11]
Coverage dose 1	93–98%	90%	87%	EPI assumptions
Dropout 1^st^ to 2^nd^ dose*	3–12%	5%	10%	
Dropout 2^nd^ to 3^rd^ dose*	3–5%	5%	0%	
Dropout 3^rd^ to 4^th^ dose*	9–50%	10%	35%	
**Service delivery**				
Proportion vaccinated in static clinic(doses 1,2,3/dose 4)	75%/30%	100%/60%	95%/95%	EPI assumptions
Proportion vaccinated in outreach clinic (doses 1,2,3/dose 4)	25%/70%	0%/40%	5%/5%	
Time spent per vaccination	4 minutes	5 minutes	5 minutes	EPI assumptions
**Vaccine Characteristics**				
Wastage (vaccine/injection supplies)	10%/10%	10%/10%	20%/10%	EPI assumptions
Buffer stock (vaccine/safety boxes)^^^	25%/10%	25%/10%	25%/10%	EPI assumptions
**Procurement add on to vaccine cost**				
Freight, insurance, inspection	30%	15%	3.5%	EPI
Handling	4.0%	5.0%	4.5%	
Clearance charge	1.0%	1.0%	3.0%	
Other (taxes and railroad levy)	0.0%	0.0%	3.5%	

Notes: *Assumed to vary across pilot areas in Malawi given wider variation in vaccination coverage for other vaccines within the pilot area. The within pilot area vaccination coverage for other vaccines in Ghana and Kenya was less pronounced. ^Buffer stock is a % of total demand for the first year, and the % of incremental demand in the subsequent years.

### Recurrent and introduction (initial setup) costs

Recurrent costs include the value of resources incurred on an ongoing basis and that last less than one year. These include operational costs of the program, such as the value of personnel time, transport, maintenance, monitoring and evaluation, and supervision, as well as costs of short-term training activities that last less than one year. Introduction costs include costs associated with initial setup, such as costs of capital resources. Introduction costs also include non-recurring activities such as microplanning, initial training, development of materials used in social mobilization, as well as additional cold chain equipment, and other capital resources. Introduction costs or initial setup costs include the value of resources that last longer than one year and are non-recurrent costs.

### Financial and economic costs

Financial costs include the value of resources purchased by the governments for the RTS,S/AS01_E_ introduction—resources such as vaccines, injection supplies, outreach allowances and per diems, and resources used in training and developing new communication materials. Financial costs do not include costs of resources already paid for or owned by the government such as health workers’ salaries. Economic costs include all financial costs as well as the value of in-kind resources used for interventions (i.e., salaries of current health personnel, volunteer labor, donated supplies, and the opportunity cost of capital goods, where applicable). The value of donated goods and services is included in economic costs. The cost of vaccine procurement is included in the economic cost and not in financial costs in the baseline financial scenario. Any procurement-related add-on costs such as insurance, freight, etc. are included as financial costs. Discounting is only conducted for economic costs whereas annualization is done for both economic and financial costs.

### Study outcomes

The key outcomes of this analysis are the incremental cost per dose of vaccination, cost of delivery per dose of vaccination, and cost per fully vaccinated child (FVC), where a fully vaccinated child is defined as a child receiving all four doses of RTS,S/ASO1_E_. Both introduction (start-up) costs and recurrent costs were added over the analysis period and divided by the total number of expected vaccinations to estimate the cost per dose of vaccination, and by number of expected FVCs over the same period to estimate cost per FVC.

The cost per dose is estimated by dividing the total cost of the program by the total number of doses delivered for the duration of the analysis. The cost per FVC is calculated by dividing the total cost by the number of children receiving all 4 doses of RTS,S/ASO1_E_. The cost of delivery per dose is calculated by subtracting the procurement and procurement add-on costs from the total cost and dividing it by the total number of doses delivered. For each output, both financial and economic costs are estimated separately. Costs are presented in 2017 US dollars (USD).

## Results

Using the target population in the pilot implementation and comparison areas, and assuming the vaccine coverage rate provided by the governments (Table 1), we projected the total target population and the number of fully vaccinated children in each area (Table 2). Under scenario 1 (i.e., continue vaccination in pilot implementation areas), approximately 167,000, 185,000, and 170,000 children in Malawi, Ghana and Kenya respectively, would be targeted for vaccination annually. Of these, about 60%, 56%, and 39% of children in Malawi, Ghana, and Kenya, respectively, are projected to be fully vaccinated with all four doses of the malaria vaccine. Under scenario 2, where malaria vaccine introduction would be expanded to comparison areas in addition to continued vaccination in the implementation areas, the target population for vaccination as well as the number of FVCs roughly doubles (See Table 2).

**Table 2:** Target population and fully vaccinated children for seven years (2020–2026).

Metric	Malawi		Ghana		Kenya	
	Total	Average annual	Total	Average annual	Total	Average annual
Scenario 1: Continue vaccination in MVIP implementation areas only						
Surviving infants targeted for vaccination	1,168,740	166,924	1,297,977	185,425	1,194,336	170,619
Projected number of vaccinations	3,892,247	556,035	4,008,686	572,669	3,329,921	475,703
Fully vaccinated children	693,230	99,033	731,336	104,477	468,547	66,937
Scenario 2: Continue vaccination in MVIP implementation and expand to comparison areas						
Surviving infants targeted for vaccination	2,195,693	313,670	2,562,387	366,055	2,351,601	335,943
Projected number of vaccinations	7,314,583	1,044,940	7,852,650	1,121,807	6,556,481	936,640
Fully vaccinated children	1,303,953	186,279	1,430,334	204,333	922,550	131,793

The incremental financial and economic cost estimates are provided in Table 3. Under the baseline price assumption, where the government pays none of the vaccine procurement costs (but pays procurement add-on costs), the financial cost per dose of vaccination is estimated to be $2.44 in Malawi, $2.28 in Ghana, and $1.78 in Kenya. The cost of delivering a dose of vaccine, excluding procurement add-on costs, is estimated to be $0.24 in Malawi, $0.90 in Ghana, and $0.71 in Kenya. The financial cost per FVC is estimated to be $13.69 in Malawi, $12.49 in Ghana, and $12.66 in Kenya.

**Table 3:** Unit costs of continuing to vaccinate in pilot areas (in 2017 USD).

Metric	% of procurement cost paid by government	Malawi		Ghana		Kenya	
		Financial	Economic	Financial	Economic	Financial	Economic
Scenario 1: Continue vaccination in MVIP implementation areas only							
Cost per dose	0%	2.44	8.24	2.28	8.73	1.78	8.46
	50%	4.30	NA	4.90	NA	4.43	NA
	100%	8.15	NA	7.80	NA	7.98	NA
Cost of delivery per dose	NA	0.24	0.33	0.90	1.66	0.71	1.19
Cost per FIC	0%	13.69	46.29	12.49	47.87	12.66	60.12
	50%	24.11	NA	27.03	NA	31.50	NA
	100%	45.77	NA	42.66	NA	56.73	NA
Scenario 2: Continue vaccination in MVIP implementation and expand to comparison areas							
Cost per dose	0%	2.42	8.22	2.09	8.42	1.70	8.37
	50%	4.28	NA	5.05	NA	4.42	NA
	100%	8.13	NA	7.90	NA	7.97	NA
Cost of delivery per dose	NA	0.23	0.32	0.72	1.34	0.63	1.10
Cost per FIC	0%	13.58	46.14	11.50	46.22	12.09	59.47
	50%	24.00	NA	27.72	NA	31.41	NA
	100%	45.63	NA	43.38	NA	56.64	NA

Similarly, the economic unit cost per dose of vaccination is estimated roughly four times higher than the financial cost at $8.24 in Malawi, $8.73 in Ghana, and $8.46 in Kenya. The economic cost per FVC is estimated at $46.29 in Malawi, $47.87 in Ghana, and $60.12 in Kenya. The economic cost per dose of delivery, excluding procurement add-on costs, is lowest in Malawi at $0.33 per dose, followed by $1.19 in Kenya, and $1.66 Ghana.

Under alternative scenarios that assume governments pay for 50% and 100% of vaccine cost, the financial cost per dose of vaccination estimate increases to $4.30 and $8.15 in Malawi, $4.90 and $7.80 in Ghana, and $4.34 and $7.98 in Kenya, respectively. The financial cost per FVC increases to $24.11 and $45.77 in Malawi, to $27.03 and $42.66 in Ghana, and $31.50 and $56.73 in Kenya.

All unit cost estimates under scenario 2 are slightly lower than scenario 1 (see Table 3). This is because the initial setup costs especially related to the activities such as national level training and sensitization, were distributed over a larger denominator of target population in scenario 2 compared to scenario 1.

### Introduction/Initial setup costs

Introduction/ initial setup costs, under scenario 1, constitute a relatively small fraction of the total financial cost in Malawi (<2%), and about 6% and 15% in Ghana and Kenya, respectively. The initial setup costs included the cost of activities such as microplanning, trainings, initial sensitization, and cold chain expansion (*only in Ghana*). In Malawi, 56% of the initial setup cost is attributed to training, 26% to microplanning, and the rest (17%) to initial sensitization activities. In Kenya, about 64% of the initial setup cost is attributed to training, followed by 28% microplanning, and <3% initial sensitization. In Ghana, about 31% of the initial setup cost is attributed to training, followed by 30% in cold chain expansion, 28% in microplanning, and <3% in initial sensitization activities. These non-recurring initial setup costs constitute an even smaller fraction of the total economic costs in all countries. Initial setup costs constitute a relatively smaller fraction of the total costs under scenario 2 compared to scenario 1 (see Supplementary material, S3 Table) as some of the introduction costs under scenario 2, especially for the continuing areas, will already have been accounted for during the pilot.

### Recurrent costs

**Fig 1:**
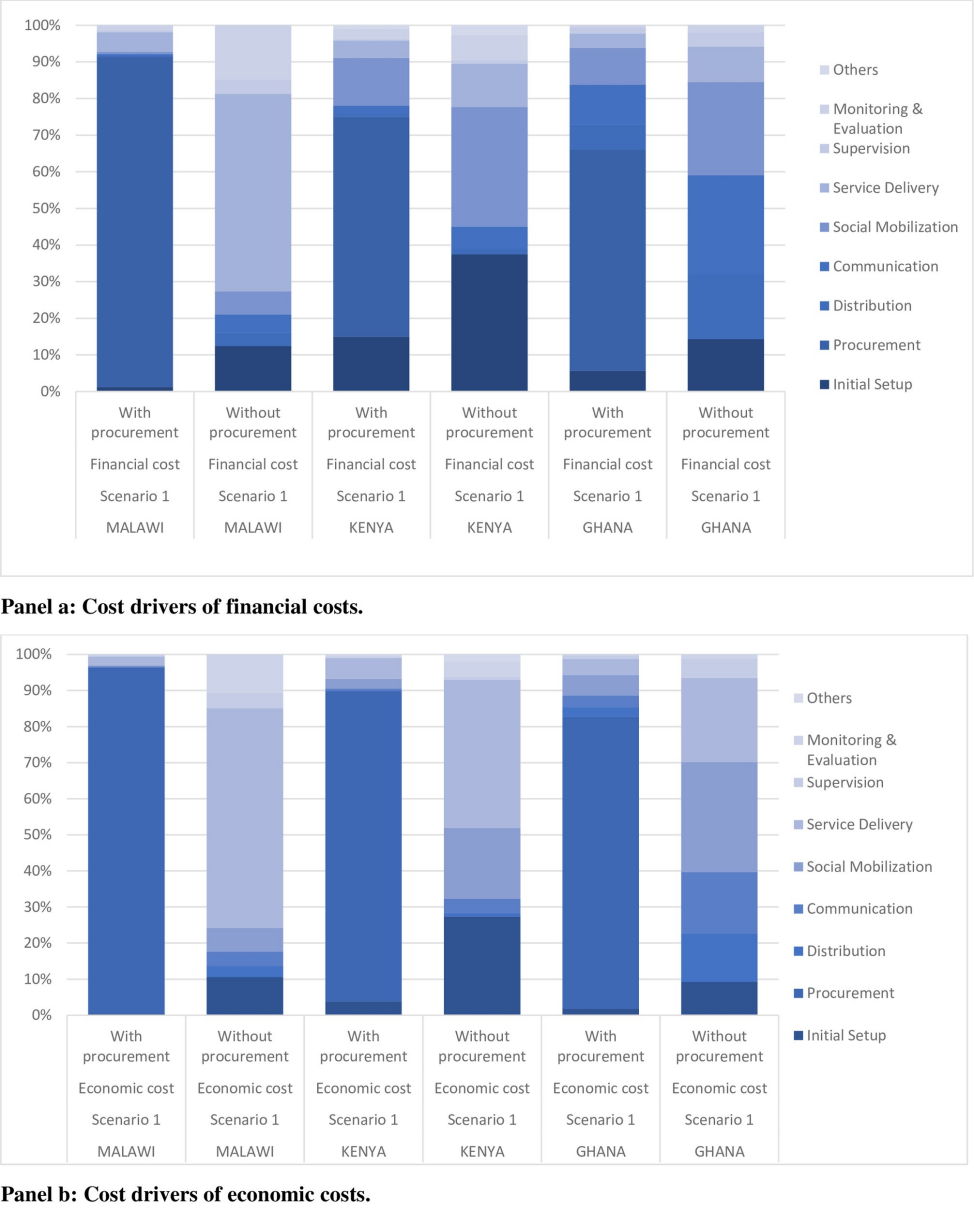
Cost drivers of continuing to vaccinate with RTS,S/ASO1_E_ in pilot intervention areas*. Panel a. Cost drivers of financial costs. Panel b. Cost drivers of economic costs. *Note: Assumes vaccines are donated (i.e., Government pays 0% of vaccine costs).

Under scenario 1, assuming the government does not pay for the vaccine costs, procurement costs constitute the major cost driver across all countries: about 60% of financial costs (which exclude vaccine costs but include procurement add-on costs) and >80% of economic cost (which includes vaccine cost and procurement add-on cost) in Ghana and Kenya, and >90% of both financial and economic costs in Malawi. Malawi shows higher procurement add-on costs than the other two countries, as it is a landlocked country. These costs constitute a single major cost driver in Malawi. Excluding all procurement-related add-on costs, the major financial cost drivers in Malawi are service delivery (53.93%), followed by monitoring and evaluation (14.73%), social mobilization (6.30%), and communications (5.02%). In Kenya, excluding procurement add-on costs, the financial cost drivers are, social mobilization (33%), service delivery (12%), and monitoring and evaluation (7%). Similarly, in Ghana, excluding procurement, the major cost drivers of the financial costs are communication (27%), social mobilization (25%), distribution (17%), and service delivery (10%) (see Fig 1).

Under scenario 2, where vaccination expands to pilot control areas, both financial and economic unit costs of vaccinating children, cost of delivering vaccine, and the cost per FVC are slightly less, although comparable to the estimates from scenario 1 (see Table 3). The cost drivers under scenario 2 are similar to those in scenario 1 (see Supplementary material, S3 Table). Differences in activities across countries lead to variation in cost drivers across countries.

## Discussion

We projected costs of continuing malaria vaccination in the pilot implementation areas to inform decisions in the three participating countries. The estimates generated provide useful insights into the cost drivers and unit costs of vaccine delivery for the pilot countries, and can be applied to RTS,S/ASO1_E_ as well as other vaccines. We refrain from extensive cross-country comparisons, as these estimates were based on country-specific inputs, assumptions, and unit costs agreed upon by the country study team in each country at the time of analysis.

Across countries, under the baseline assumption that the government does not pay for vaccine costs, our estimated incremental financial costs range from $1.70 to $2.44 per dose delivered, $0.23 to $0.72 per dose delivered (excluding procurement add-on costs), and $11.50 to $13.69 per FVC. The financial cost per FVC increases roughly three-fold if the government pays the full cost of vaccines. Estimates of economic costs per dose are between 3 and 5 times higher than financial costs and are driven mainly by the vaccine price. The estimates of unit cost per dose and cost per FVC is comparable across countries, although the cost of delivery per dose is significantly lower in Malawi compared to the others. In addition to variations in activities, the unit costs associated with per diems and allowances incurred in carrying out activities is relatively small in Malawi, contributing to lower cost estimates.

Cost of vaccine and other immunization supplies are the number one cost drivers across all countries, accounting for up to more than 90% of total financial and economic costs, similar to findings in other studies [12,13]. Even in scenarios that assume the government does not pay for the vaccine, procurement add-on costs added as a percentage of per unit product cost contributes to a large proportion of the overall program cost. For example, in Malawi, the procurement add-on costs are assumed to be 35%, the largest among all countries, driving the overall procurement costs very high for a four-dose vaccine and contributing to a large difference between the cost per dose and cost of delivery per dose. Minimizing the procurement add-on costs to the government, for example through donor subsidies, or local policy changes, such as reducing local taxes on imports, would significantly lower the financial cost per dose across countries and particularly in Malawi.

A few other studies have estimated the cost of delivering RTS,S/AS01_E_ in sub-Saharan Africa including Ghana and Kenya. Galactionova and colleagues [12] reported the estimates of cost per FVC, for a 4-dose vaccine schedule, in Ghana at $27.78 (financial) and $30.46 (economic), and in Kenya at $40.15 (financial) and $49.80 (economic), in USD 2017 units. Further, their estimate of economic cost of vaccine delivery (net of vaccine and immunization supplies) was $0.91 in Ghana, and $2.43 in Kenya [12]. Another recent study by Sicuri and colleagues [13] estimated the economic cost per FVC at $28.06 for Ghana and $40.41 for Kenya, in USD 2017 units. Their estimates of cost of delivery per dose were $0.22 in Ghana and $0.41 in Kenya [13]. Estimates of FVC from both studies are lower than our estimates where financial cost per FVC are $12.49 (Ghana) and $12.66 (Kenya), and the economic costs per FVC are $47.81 (Ghana) and $60.12 (Kenya). Our cost of delivery estimates, although not directly comparable, are within the range reported in these studies [12,13] (Ghana at $0.72- $0.90 (financial), $1.34-$1.66 (economic), and Kenya at $0.63- $0.71 (financial), $1.10-$1.19 (economic)). There are a few noteworthy key differences in assumptions and cost calculations approach across these studies that attribute to the differences in cost estimates.

Galactionova and colleagues [12] used a generic set of activities, assumptions and inputs to estimate the costs, whereas our study projected the activities adapted from the country-specific malaria vaccine plans for the pilot and are country specific [9–11]. Our study identified spare capacity for vaccine storage in two of the three countries and therefore did not include any fixed costs associated with strengthening the cold chain in those settings reflecting the actual needs in country. This is contrast to the Sicuri et al. [13], which identify, and value incremental resource needs related to introduction of vaccine. Although all studies used a base vaccine price of $5 per dose, Sicuri et al. [13] assume the base price to include vaccine wastage as well as the procurement add-on costs, while our study assumes both wastage and procurement add-on as an addition to the baseline vaccine price based on interviews in countries. Further, Sicuri et al. [13] assumed full coverage of all children, while our study assumes a different vaccine coverage rate based on the expectation from the EPI. The coverage drop-out from the third to the fourth dose is as high as 30% in Kenya, and up to 50% in some of the areas in Malawi based on other vaccine coverage estimates in respective areas from the EPI. Vaccine drop-out rate substantially contribute to the cost per FVC. Although the actual coverage and wastage are not yet known in the context of a 4-dose malaria vaccine, our estimates utilize anticipated coverage that varies by sub-regions/districts as estimated by the EPI representatives in respective countries. Further, both the Galactionova [12] and Sicuri [13] studies assumed a national roll-out while the scope of our study is limited to the malaria vaccine pilot areas only. The costs of introduction activities that occur at the national level is allocated to each child in study areas only increasing the unit cost estimates per child, reflecting the current realities in the context of pilot implementation. Moreover, our unit cost estimates are slightly lower under scenario 2 compared to scenario 1 indicating some economy of scale by expansion in comparison areas which could further be harnessed at national level scale-up. Although the initial setup costs are incurred at the national level, these activities are relatively limited and there could be cost savings if the program were to scale-up more broadly across country.

While there are limited studies available from the pilot countries to understand how costs of delivering the RTS,S/ASO1_E_ vaccine compares to existing EPI vaccines, our estimates are not substantially different from estimates for other EPI vaccines. For instance, the incremental costs per dose (excluding vaccine costs) for other EPI vaccines are reported to be $0.90 in Ghana [14], $1.51 in Kenya [15], $0.52 in Malawi [16]. Recognizing that the vaccines are different and may require slightly different approaches and delivery strategies, it is encouraging to note that cost of delivery (excluding vaccine cost) for the malaria vaccine in these countries is not widely different from other studies. This may suggest that in the future, once a “sufficient” pool of cost of delivery estimates is available, it could potentially be leveraged to infer insights on new vaccine introduction delivery costs by national programs.

Our financial cost estimates constitute a significant proportion of the annual budget of immunization programs in the pilot countries, in particular cost estimates including vaccine cost. The financial cost under scenario 1 (continuing RTS,S/ASO1_E_ vaccination in the MVIP implementation areas), assuming the government does not pay/pays 50%/pays 100% for vaccine costs, ranged from 0.51%/1.27%/2.29% of the total average annual cost of all immunization services in Kenya to 2.46%/4.90%/8.64% of the same in Ghana, and 3.26%/5.74%/10.90% in Malawi [17–19]. Similarly, the cost estimates under scenario 2 (introduction in both implementation and comparison areas) amount to between 0.96%/2.50%/4.51% in Kenya, 4.44%/9.21%/16.53% in Ghana, and 6.08%/10.75%/20.44% in Malawi of the average annual cost of the national immunization program. Although we do not discuss the potential sources of co-financing for the purchase or provision of the vaccine under any of the outlined scenarios, the range of estimates under different cost sharing plans for vaccines that may be assumed by the governments of the respective countries would provide a range of potential cost implications for them. Further, the financing implications should be considered in the context of potential cost savings on malaria treatment and care due to the vaccine’s impact on reducing malaria disease burden. Quantifying this is beyond the scope of this analysis.

One of the strengths of this paper is that the cost estimates included in this study are generated based on country-specific inputs and assumptions of activities and unit costs, reflecting variations in program implementation across countries. The context-specific activities and assumptions, although realistic to the local context, make results less generalizable and limits comparability cross-country. The results from this analysis will require careful interpretation when making inferences about these or other program costs.

This study has several limitations. Our analysis assumed an initial completion of the pilot implementation by 2020 and began the costing of continued vaccination, starting year 2020. However, the implementation was delayed to 2019, and the pilot’s current completion date is 2022. Our analysis’s consideration of 2020 as a starting point for continued vaccination will likely have minimal or no impact on the cost estimates derived in this study, and the results are largely indicative of the expected costs of continuing the RTS,S/AS01_E_ program under the assumptions utilized. Further, the cost projections from this study will need to be evaluated against costs measured during the implementation of the malaria vaccine during the pilot [9–11]. Additionally, our scope in analysis is limited to pilot areas only. A national roll-out of the malaria vaccine introduction needs further analysis, but the current analysis serves as a starting point by providing the unit cost estimates for future work. Assumptions on spare capacity in the system, although true to pilot areas and thus for the current scope, may or may not hold for a full country scale-up.

## Conclusion

In summary, our analysis represents an effort to understand the cost of malaria vaccine delivery to inform country decision-makers in the pilot countries, as they face important questions on whether or not to continue malaria vaccination, should the intervention receive a positive recommendation for broader use. This analysis also adds to the evidence available on vaccine delivery costs for products delivered outside the standard immunization schedule. Notably, cost estimates from this analysis do not appear to markedly differ from other estimates for other vaccines in the same countries. This analysis also highlights important cost drivers for vaccine delivery, some of which might be influenced by global and country-specific financing and existing procurement mechanisms. Ultimately, these cost estimates will inform the financial and economic impact of expanding the provision of malaria vaccines in pilot countries and elsewhere.

## Supporting information

S1 Table List of activities identified for costing malaria vaccine introduction and delivery. (DOCX)

S2 Table Activities categorized by type of cost. (DOCX)

S3 Table Total costs (for 7 years) and cost share estimates, by scenario. (DOCX)
