# Correction: Costs of continuing RTS,S/ASO1E malaria vaccination in the three malaria vaccine pilot implementation countries

**DOI:** 10.1371/journal.pone.0250863

**Published:** 2021-04-22

**Authors:** Ranju Baral, Ann Levin, Chris Odero, Clint Pecenka, Collins Tabu, Evans Mwendo, George Bonsu, John Bawa, John Frederick Dadzie, Joyce Charo, Kwadwo Odei Antwi-Agyei, Kwame Amponsa-Achianou, Rose Eddah Jalango, Rouden Mkisi, Scott Gordon, Temwa Mzengeza, Winthrop Morgan, Farzana Muhib

Cost estimates under additional vaccine price assumptions are not included in the main result Table (Table 3). Please view S4 Table, “Unit cost of continuing to vaccinate in pilot areas at various vaccine prices,” for results under additional vaccine price assumptions below.

In the Procurement subsection of the Materials and methods, there is information missing in the fourth sentence. The correct sentence is: The cost of the vaccine was assumed to vary between $2, $5, and $10 per dose as there is no marked price for the vaccine due to uncertain demand. Detailed results are presented only for $5 per dose in the main text for simplicity. Additional estimates for other price assumptions are included in the supplementary material (S4 Table).

## Supporting information

S4 Table Unit cost of continuing to vaccinate in pilot areas at various vaccine prices. (DOCX)
