# Supplementary material for: Costs of continuing RTS,S/ASO1E malaria vaccination in the three malaria vaccine pilot implementation countries
Source: PLoS One. 2021 Jan 11;16(1):e0244995. doi: 10.1371/journal.pone.0244995 (PMC7799756; doi:10.1371/journal.pone.0244995)
Supplement: S1 Table — (DOCX) [file pone.0244995.s001.docx]

**S1 Table. List of activities identified for costing malaria vaccine introduction and delivery.**

| **Malawi** | | | **Ghana** | | | **Kenya** | |
| --- | --- | --- | --- | --- | --- | --- | --- |
| **Microplanning** | | |  | | |  | |
| - National level planning meeting for microplanning - National level micro-plan aggregation meeting - District microplanning and aggregation of micro-plans from health facilities - Health facility microplanning data collection | | | - Preparations for microplanning at national level - Preparations for microplanning at regional level - District level microplanning | | | - Health facility microplanning meetings | |
| **Procurement** | | |  | | |  | |
| - Purchase of vaccines and injection supplies including shipping and related costs (procurement add-on costs) | | | - Purchase of vaccines and injection supplies including shipping and related costs (procurement add-on costs) | | | - Purchase of vaccines and injection supplies including shipping and related costs (procurement add-on costs) | |
| **Distribution** | | |  | | |  | |
| - Quarterly supply trips to the national vaccine store from each regional storage site - Monthly supply trips from districts to a regional store - Monthly supply trips from district stores to health facilities | | | - Quarterly supply trips from central stores to a regional store - Monthly supply trips from regional stores to district store - Monthly supply trips from district to health facilities | | | - Quarterly supply trips from central stores to a regional store - Semi-annual injection supplies from national stores to county stores - Monthly vaccine/injection supply distribution trip from county to sub-county - Monthly supply trips from sub-county to health facilities | |
| **Training** | | |  | | |  | |
| - Training material development (includes communication materials and job-aides revision) - Planning meeting for training - Training of trainers - Training of service providers/health workers | | | - Training of trainers at the national level - Trainings of supervisory teams at regional level - District training of service delivery personnel | | | - Refresher training of trainers - Training of county level trainers - Training of trainers at sub-county level - Training of service providers from health facilities | |
| **Sensitization** | |  | | |  | |  |
| - National level stakeholders briefing - Media preparation and sensitization - Community leader’s sensitization - District health management team sensitization meetings - District executive committee sensitization meeting | | - Briefing of stakeholders (traditional leaders, Representatives of religious bodies, Ghana Journalists Association, pediatric soc. of Ghana, Ghana Medical Association) - Regional Press briefing - Sensitization meetings with district leaders | | | - Stakeholders meeting at national Level - Press briefing - Health professional engagement - County level sensitization event - Sensitization meetings with sub-County Leaders including community health focal person | |  |
| **Social mobilization** | |  | | |  | |  |
| - Peer educator mobilization - Counselor and volunteer mobilization - Support meeting for community leaders | | - Social mobilization in CWC catchment areas (Community durbars) - Follow-up mobilization in CWC catchment areas - RTS,S/AS01_E_ - 4 Household Visits by Volunteers | | | - Radio spots - Health talks-community leader (Barazas) awareness meeting community level - Support meeting for community health volunteers at health facility level | |  |
| **Communication** | |  | | |  | |  |
| - Press release - National and district level spokespersons preparation workshop - Printings, job-aides, posters, and leaflets | | - Printings, information material package print run (patient) - Printings, job-aids for service delivery personnel - Production and broadcast of mass media public service announcements | | | - Printings, flip chart, flyer, key fact booklet, poster - Printings, job aids for social mobilizers / vaccinators - Spokesperson and crisis communication workshop at national/county/sub-county levels | |  |
| **Service delivery** | |  | | |  | |  |
| - Administer RTS,S/ASO1 _E_ vaccines during a routine fixed clinic session - Administer RTS,S/ASO1 _E_ vaccines during routine outreach | | - Administer RTS,S/ASO1 _E_ vaccines during a routine fixed and outreach session - Administer vaccines during a special RTS/ASO1 campaign activity (or mop-up) | | | - Administer RTS,S/ASO1 _E_ vaccines during a routine fixed clinic session - Administer RTS,S/ASO1 _E_ vaccines during routine outreach | |  |
| **Supervision** |  | | |  | | |  |
| - Pre-introduction team supervision (from national, regional levels) - Post introduction team supervision (from national, regional levels - Quarterly supportive supervision from national level - Quarterly supportive supervision visit from district level | - Supportive supervision visit from district EPI supervisory team | | | - Supervisory visit from national level after introduction - Biannual supportive supervision from national level - Supervisory visit from county level after introduction - Quarterly supportive supervision visit from sub-county to health facility | | |  |
| **Monitoring and evaluation** |  | | |  | | |  |
| - Print stickers for record keeping - Print child health passports - Health facility monitoring tools package (support) | - Modification of EPI Monitoring Tool (by National) - National quarterly review of RTS,S/AS01 Program - Regional annual review of RTS,S/AS01 Program | | | - Print stickers for record keeping - Print mother child book/ passports - National and county coordination monitoring/supervision during introduction (readiness assessment) - Annual monitoring record supply package (tally sheets, summary sheets, ledger) | | |  |
| **Others** |  | | |  | | |  |
| - None | - None | | | - AEFI case investigation - (assume six case per year) - NITAG meeting- quarterly - Kenya national vaccine safety advisory committee (KNVSAC) quarterly meeting | | |  |
| **Cold chain expansion** |  | | |  | | |  |
| - None | - Purchase of additional cold chain equipment: - National level: 1 walk in cold room - District level: 20 units AC; 76 units cold boxes - Facility level: 185 units cold boxes | | | - None | | |  |
