# Supplementary material for: Costs of continuing RTS,S/ASO1E malaria vaccination in the three malaria vaccine pilot implementation countries
Source: PLoS One. 2021 Jan 11;16(1):e0244995. doi: 10.1371/journal.pone.0244995 (PMC7799756; doi:10.1371/journal.pone.0244995)
Supplement: S2 Table — (DOCX) [file pone.0244995.s002.docx]

**S2 Table. Activities categorized by type of cost.**

| Activities | Recurrent Costs | | Initial set up costs | |
| --- | --- | --- | --- | --- |
|  | Financial | Economic | Financial | Economic |
| Procurement |  |  |  |  |
|  | - Vaccines & injection supplies purchased by government. - Freight, clearance, insurance, taxes | - Vaccines & injection supplies any source financing - Freight, clearance, insurance, taxes |  |  |
| Distribution |  |  |  |  |
|  | - Driver per diems - Transport fuel | - Driver time/per diems - Transport fuel | - Vehicle purchased by government | - Vehicle purchase |
| **Microplanning** |  |  |  |  |
|  |  |  | - Per diems/ allowances - Venue rental - Transport | - Personnel time in meetings - Per diems/ allowances - Venue rental - Transport |
| **Training** |  |  |  |  |
| *Capital cost if preparatory training* | - Refresher training by government: - Per diems and travel allowances - Venue rental - Transport - Training Materials - Stationery | - Refresher training by donor: - Per diems and travel allowances - Venue rental - Transport - Training Materials - Stationery | - Preparatory training: - Training material dev. - Per diems and travel allowances - Venue rental - Transport - Training Materials - Stationery | - Preparatory training: - Personnel time spent on training - Training material dev - Per diems and travel allowances - Venue rental - Transport - Training Materials - Stationery |
| **Sensitization, Social Mobilization, Communication** | | | | |
|  |  |  | - Facilitator time in meetings - Per diems and travel allowances - Stationery - Printing of materials - Production of TV and/or radio spots | - Value of personnel, and volunteer time spent on material development and other activities - Facilitator time in meetings - Per diems and travel allowances - Stationery - Printing of posters and leaflets - Production of TV and/or radio spots |
| **Service delivery** | | | | |
|  | - Value of personnel time spent on vaccination - Transport fuel - Personnel per diems to travel to vaccination sites |  |  |  |
| **Supervision, Monitoring and Evaluation** | | | | |
|  | - Personnel time spent on supervision, monitoring, and eval. - Travel allowances - Transport fuel and maintenance - Stationery |  |  |  |
| **Cold Chain Expansion** | | | | |
|  |  |  | - Cold chain equipment (annualized) | - Cold chain equipment (annualized and discounted) |
