# Supplementary material for: Costs of continuing RTS,S/ASO1E malaria vaccination in the three malaria vaccine pilot implementation countries
Source: PLoS One. 2021 Jan 11;16(1):e0244995. doi: 10.1371/journal.pone.0244995 (PMC7799756; doi:10.1371/journal.pone.0244995)
Supplement: S3 Table — (DOCX) [file pone.0244995.s003.docx]

**S3 Table: Total costs (for 7 years) and cost share estimates, by scenario.**

*Country: Malawi*

| **Activity group** | **Scenario 1** | | | | | | **Scenario 2** | | | | | |
| --- | --- | --- | --- | --- | --- | --- | --- | --- | --- | --- | --- | --- |
|  | **Financial cost** | | | **Economic cost** | | | **Financial cost** | | | **Economic cost** | | |
|  | Total cost | % of total cost | % of total cost w/o proc. | Total cost | % of total cost | % of total cost w/o proc. | Total cost | % of total cost | % of total cost w/o proc. | Total cost | % of total cost | % of total cost w/o proc. |
| **Initial Setup** | **117,210** | **1.24%** | **12.43%** | **137,900** | **0.43%** | **10.60%** | **127,214** | **0.72%** | **7.71%** | **155,528** | **0.26%** | **6.74%** |
| Microplanning | 30,789 | 0.32% | 3.27% | 38,723 | 0.12% | 2.98% | 33,205 | 0.19% | 2.01% | 44,723 | 0.07% | 1.94% |
| Training | 65,917 | 0.69% | 6.99% | 68,783 | 0.21% | 5.29% | 65,917 | 0.37% | 3.99% | 68,783 | 0.11% | 2.98% |
| Initial sensitization | 20,503 | 0.22% | 2.18% | 30,394 | 0.09% | 2.34% | 28,091 | 0.16% | 1.70% | 42,022 | 0.07% | 1.82% |
| Cold chain expansion | 0 | 0.00% | 0.00% |  | 0.00% | 0.00% |  | 0.00% | 0.00% |  | 0.00% | 0.00% |
| Procurement | 8,547,393 | 90.07% |  | 30,784,833 | 95.94% |  | 16,062,888 | 90.68% |  | 57,853,108 | 96.16% |  |
| Distribution | 33,785 | 0.36% | 3.58% | 39,149 | 0.12% | 3.01% | 51,086 | 0.29% | 3.09% | 58,471 | 0.10% | 2.53% |
| Communication | 47,319 | 0.50% | 5.02% | 52,381 | 0.16% | 4.02% | 71,443 | 0.40% | 4.33% | 76,506 | 0.13% | 3.31% |
| Social Mobilization | 59,348 | 0.63% | 6.30% | 85,968 | 0.27% | 6.61% | 117,597 | 0.66% | 7.12% | 170,344 | 0.28% | 7.38% |
| Service Delivery | 508,346 | 5.36% | 53.93% | 792,578 | 2.47% | 60.90% | 955,578 | 5.39% | 57.88% | 1,489,764 | 2.48% | 64.51% |
| Supervision | 37,827 | 0.40% | 4.01% | 54,682 | 0.17% | 4.20% | 66,074 | 0.37% | 4.00% | 96,734 | 0.16% | 4.19% |
| Monitoring & Evaluation | 138,816 | 1.46% | 14.73% | 138,816 | 0.43% | 10.67% | 261,833 | 1.48% | 15.86% | 261,833 | 0.44% | 11.34% |
| Others | 0 | 0.00% | 0.00% | - | 0.00% | 0.00% | - | 0.00% | 0.00% | - | 0.00% | 0.00% |
| **Total cost (in USD)** | **9,490,043** | **1** | **1** | **32,086,307** | **1** | **1** | **17,713,712** | **1** | **1** | **60,162,288** | **1** | **1** |

*Country: Kenya*

| **Activity group** | **Scenario 1** | | | | | | **Scenario 2** | | | | | |
| --- | --- | --- | --- | --- | --- | --- | --- | --- | --- | --- | --- | --- |
|  | **Financial cost** | | | **Economic cost** | | | **Financial cost** | | | **Economic cost** | | |
|  | Total cost | % of total cost | % of total cost w/o proc. | Total cost | % of total cost | % of total cost w/o proc. | Total cost | % of total cost | % of total cost w/o proc. | Total cost | % of total cost | % of total cost w/o proc. |
| **Initial Setup** | **887,120** | **14.96%** | **37.44%** | **1,083,065** | **3.84%** | **27.37%** | **1,538,959** | **13.79%** | **37.12%** | **1,889,102** | **3.44%** | **26.27%** |
| Microplanning | 254,914 | 4.30% | 10.76% | 318,899 | 1.13% | 8.06% | 492,359 | 4.41% | 11.88% | 615,944 | 1.12% | 8.56% |
| Training | 574,585 | 9.69% | 24.25% | 703,046 | 2.50% | 17.77% | 988,980 | 8.86% | 23.85% | 1,212,037 | 2.21% | 16.85% |
| Initial sensitization | 57,621 | 0.97% | 2.43% | 61,121 | 0.22% | 1.54% | 57,621 | 0.52% | 1.39% | 61,121 | 0.11% | 0.85% |
| Cold chain expansion |  | 0.00% | 0.00% |  | 0.00% | 0.00% |  | 0.00% | 0.00% |  | 0.00% | 0.00% |
| Procurement | 3,560,512 | 60.04% |  | 24,212,202 | 85.95% |  | 7,010,550 | 62.84% |  | 47,672,980 | 86.89% |  |
| Distribution | 37,232 | 0.63% | 1.57% | 37,232 | 0.13% | 0.94% | 61,072 | 0.55% | 1.47% | 61,072 | 0.11% | 0.85% |
| Communication | 142,755 | 2.41% | 6.02% | 159,422 | 0.57% | 4.03% | 143,787 | 1.29% | 3.47% | 160,454 | 0.29% | 2.23% |
| Social Mobilization | 773,346 | 13.04% | 32.64% | 773,346 | 2.75% | 19.54% | 1,472,902 | 13.20% | 35.53% | 1,472,902 | 2.68% | 20.48% |
| Service Delivery | 281,641 | 4.75% | 11.89% | 1,627,598 | 5.78% | 41.13% | 554,541 | 4.97% | 13.38% | 3,204,675 | 5.84% | 44.56% |
| Supervision | 22,091 | 0.37% | 0.93% | 29,919 | 0.11% | 0.76% | 22,091 | 0.20% | 0.53% | 29,919 | 0.05% | 0.42% |
| Monitoring & Evaluation | 163,950 | 2.76% | 6.92% | 170,964 | 0.61% | 4.32% | 291,226 | 2.61% | 7.02% | 298,240 | 0.54% | 4.15% |
| Others | 61,302 | 1.03% | 2.59% | 75,559 | 0.27% | 1.91% | 61,302 | 0.55% | 1.48% | 75,559 | 0.14% | 1.05% |
| **Total cost (in USD)** | **5,929,949** | **1** | **1** | **28,169,307** | **1** | **1** | **11,156,431** | **1** | **1** | **54,864,902** | **1** | **1** |

*Country: Ghana*

| **Activity group** | **Scenario 1** | | | | | | **Scenario 2** | | | | | | |
| --- | --- | --- | --- | --- | --- | --- | --- | --- | --- | --- | --- | --- | --- |
|  | **Financial cost** | | | **Economic cost** | | | **Financial cost** | | | | **Economic cost** | | |
|  | Total cost | % of total cost | % of total cost w/o proc. | Total cost | % of total cost | % of total cost w/o proc. | Total cost | % of total cost | % of total cost w/o proc. | Total cost | | % of total cost | % of total cost w/o proc. |
| **Initial Setup** | **519,369** | **5.69%** | **14.32%** | **614,395** | **1.76%** | **9.26%** | **826,210** | **5.02%** | **14.61%** | **1,011,046** | | **1.53%** | **9.61%** |
| Microplanning | 148,772 | 1.63% | 4.10% | 193,492 | 0.55% | 2.92% | 231,755 | 1.41% | 4.10% | 312,836 | | 0.47% | 2.97% |
| Training | 161,043 | 1.76% | 4.44% | 207,317 | 0.59% | 3.12% | 349,536 | 2.13% | 6.18% | 440,164 | | 0.67% | 4.18% |
| Initial sensitization | 50,444 | 0.55% | 1.39% | 54,476 | 0.16% | 0.82% | 85,809 | 0.52% | 1.52% | 98,936 | | 0.15% | 0.94% |
| Cold chain expansion | 159,110 | 1.74% | 4.39% | 159,110 | 0.45% | 2.40% | 159,110 | 0.97% | 2.81% | 159,110 | | 0.24% | 1.51% |
| Procurement | 5,506,451 | 60.29% |  | 28,370,871 | 81.04% |  | 10,789,372 | 65.62% |  | 55,590,732 | | 84.09% |  |
| Distribution | 639,767 | 7.00% | 17.64% | 883,136 | 2.52% | 13.31% | 1,089,070 | 6.62% | 19.26% | 1,562,314 | | 2.36% | 14.85% |
| Communication | 982,402 | 10.76% | 27.09% | 1,138,094 | 3.25% | 17.15% | 1,423,784 | 8.66% | 25.18% | 1,723,194 | | 2.61% | 16.38% |
| Social Mobilization | 924,526 | 10.12% | 25.49% | 2,022,558 | 5.78% | 30.47% | 1,284,609 | 7.81% | 22.72% | 2,432,647 | | 3.68% | 23.12% |
| Service Delivery | 350,386 | 3.84% | 9.66% | 1,545,394 | 4.41% | 23.28% | 685,906 | 4.17% | 12.13% | 3,026,246 | | 4.58% | 28.76% |
| Supervision | 141,870 | 1.55% | 3.91% | 351,369 | 1.00% | 5.29% | 275,874 | 1.68% | 4.88% | 683,258 | | 1.03% | 6.49% |
| Monitoring & Evaluation | 68,269 | 0.75% | 1.88% | 81,947 | 0.23% | 1.23% | 68,269 | 0.42% | 1.21% | 81,947 | | 0.12% | 0.78% |
| Others | - | 0.00% | 0.00% |  | 0.00% | 0.00% |  | 0.00% | 0.00% | - | | 0.00% | 0.00% |
| **Total cost (in USD)** | **9,133,040** | **1** | **1** | **35,007,765** | **1** | **1** | **16,443,095** | **1** | **1** | **66,111,385** | | **1** | **1** |
