# Supplementary material for: Correction: Costs of continuing RTS,S/ASO1E malaria vaccination in the three malaria vaccine pilot implementation countries
Source: PLoS One. 2021 Apr 22;16(4):e0250863. doi: 10.1371/journal.pone.0250863 (PMC8061918; doi:10.1371/journal.pone.0250863)
Supplement: S4 Table — (DOCX) [file pone.0250863.s001.docx]

**S4 Table.** **Unit cost of continuing to vaccinate in pilot areas at various vaccine prices.**

| **Metric** | **Assumed vaccine price per dose** | **Malawi** | | **Ghana** | | **Kenya** | |
| --- | --- | --- | --- | --- | --- | --- | --- |
|  |  | **Financial** | **Economic** | **Financial** | **Economic** | **Financial** | **Economic** |
| Scenario 1: Continue vaccination in MVIP implementation areas only | | | | | | | |
| Cost per dose | $2 | 1.24 | 3.62 | 1.56 | 4.59 | 1.24 | 4.20 |
|  | $5 | 2.44 | 8.24 | 2.28 | 8.73 | 1.78 | 8.46 |
|  | $10 | 4.44 | 15.96 | 3.48 | 15.63 | 2.68 | 15.56 |
| Cost of delivery per dose | $2 | 0.24 | 0.33 | 0.90 | 1.66 | 0.71 | 1.19 |
|  | $5 | 0.24 | 0.33 | 0.90 | 1.66 | 0.71 | 1.19 |
|  | $10 | 0.24 | 0.33 | 0.90 | 1.66 | 0.71 | 1.19 |
| Cost per FIC | $2 | 6.95 | 20.30 | 8.55 | 25.17 | 8.82 | 29.84 |
|  | $5 | 13.69 | 46.29 | 12.49 | 47.87 | 12.66 | 60.12 |
|  | $10 | 24.92 | 89.59 | 19.05 | 85.7 | 19.05 | 110.59 |
| Scenario 2: Continue vaccination in MVIP implementation and comparison areas | | | | | | | |
| Cost per dose | $2 | 1.22 | 3.60 | 1.38 | 4.28 | 1.16 | 4.11 |
|  | $5 | 2.42 | 8.22 | 2.09 | 8.42 | 1.70 | 8.37 |
|  | $10 | 4.42 | 15.94 | 3.29 | 15.32 | 2.60 | 15.47 |
| Cost of delivery per dose | $2 | 0.23 | 0.32 | 0.72 | 1.34 | 0.63 | 1.10 |
|  | $5 | 0.23 | 0.32 | 0.72 | 1.34 | 0.63 | 1.10 |
|  | $10 | 0.23 | 0.32 | 0.72 | 1.34 | 0.63 | 1.10 |
| Cost per FIC | $2 | 6.85 | 20.18 | 7.55 | 23.48 | 8.26 | 29.19 |
|  | $5 | 13.58 | 46.14 | 11.50 | 46.22 | 12.09 | 59.47 |
|  | $10 | 24.80 | 89.40 | 18.07 | 84.12 | 18.48 | 109.94 |

*Assumes % of procurement cost paid by Government at 0%.*
